# A Risk Score Signature Consisting of Six Immune Genes Predicts Overall Survival in Patients with Lower-Grade Gliomas

**DOI:** 10.1155/2022/2558548

**Published:** 2022-02-11

**Authors:** Yuxi Wu, Zesheng Peng, Sujie Gu, Haofei Wang, Wei Xiang

**Affiliations:** Department of Neurosurgery, Union Hospital, Tongji Medical College, Huazhong University of Science and Technology, Wuhan 430022, China

## Abstract

**Background:**

Lower-grade gliomas (LGGs) are less aggressive with a long overall survival (OS) time span. Because of individualized genomic features, a prognostic system incorporating molecular signatures can more accurately predict OS.

**Methods:**

Differential expression analysis between LGGs and normal tissues was performed using the Gene Expression Omnibus (GEO) datasets (GSE4290 and GSE12657). Immune-related differentially expressed genes (ImmPort-DEGs) were analyzed for functional enrichment. The least absolute shrinkage and selection operator (LASSO) analysis was performed to develop an immune risk score signature (IRSS). We extracted information from the Cancer Genome Atlas (TCGA) and the Chinese Glioma Genome Atlas (CGGA) to establish and validate the model. The relationship of model gene sets with immune infiltration was analyzed based on gene set variation analysis (GSVA) scores. Patients were divided into low- and high-risk groups based on the median score. The time-dependent receiver-operating characteristic (ROC) curve and the Kaplan-Meier curve were used to evaluate the model. Then, a precise prognostic nomogram was established, and its efficacy was verified.

**Results:**

A total of 18 related immune genes were identified, building a 6-gene IRSS (BMP2, F2R, FGF13, PCSK1, PRKCB, and PTGER3). DEGs were enriched in T cell and NK cell regulatory pathways. Immune infiltration analysis confirmed that the gene signature correlated with a decrease in innate immune cells. In terms of model evaluation, ROC curves at 1, 3, and 5 years showed moderate predictive ability of IRSS (AUC = 0.930, 0.797, and 0.728). The Cox regression analysis revealed that IRSS was an independent prognostic factor, and the nomogram model had good predictive ability (C − index = 0.828). Meanwhile, the predictive power of IRSS was also confirmed in the training cohort. The Kaplan-Meier results showed that the prognosis of the high-risk group was significantly worse in all cohorts.

**Conclusion:**

IRSS may serve as a novel survival prediction tool in the classification of LGG patients.

## 1. Introduction

Adult gliomas are the most prevalent central nervous system tumors, accounting for 75% of all primary intracranial malignancies [1]. Gliomas are graded I-IV by the World Health Organization (WHO) based on clinical symptoms and molecular marker status [2]. Lower-grade gliomas (LGGs) are defined as WHO grade II and III gliomas combined. Although the course of glioma patients varies, most gliomas, especially LGGs, can evolve progressively into glioblastoma (GBM), and molecular biomarkers are used to refine the branching of gliomas [3, 4]. Despite considerable advances in our understanding of gliomas, such as isocitrate dehydrogenase (IDH) [5] and methylguanine methyltransferase (MGMT) [6], the survival time of LGG patients remains highly variable [1]. Therefore, favorable treatment options remain inadequate.

Glioma therapeutic approaches are continually being researched, and immunotherapy may be indicated as a viable glioma treatment strategy. In the case of glioma, monoclonal antibodies, dendritic cell vaccines, and innate immune cells have all been found to have potential uses [7]. However, due to specific variances in glioma patients' immune states, the prognosis of glioma patients is difficult to predict [2, 8]. Hence, dependable strategies for identifying patient groups that may be responsive to further immunotherapy are required. Various mRNA sequence-based survival prediction indicators have turned out to be effective survival strategies [9–12]. So, an integrated immune gene risk assessment method might be useful for LGG prognosis prediction.

A simple and feasible prognostic indicator is more valuable in clinical applications [13]. One study constructed a genetic signature sensitive to IDH mutation status that could effectively predict survival in patients with groin cancer [11]. However, insufficient sample size included and available clinical data led to a bias in the ability of practical application. We obtained microenvironment-related immune genes to develop an immune prognostic risk profile based on six genes and combined them with clinical information from The Cancer Genome Atlas (TCGA) to build a prediction model. More importantly, we applied two larger sequencing cohorts from the Chinese Glioma Genome Atlas (CGGA) [14] database to validate this association. The immune risk score profile established in this study is robust, and it can be used for prognostic prediction of LGG patients to provide an accurate assessment for clinical treatment.

## 2. Material and Methods

### 2.1. Datasets

Data from 513 lower-grade glioma samples (TCGA-LGG), including mRNA sequencing data, clinical information, and survival information, were downloaded from the GlioVis network (http://gliovis.bioinfo.cnio.es/). Two CGGA (http://www.cgga.org.cn/) datasets were collected as validation sets, containing 325 and 693 tumor samples, respectively. Differential analysis was performed on GSE4290 and GSE12657 from the Gene Expression Omnibus (GEO) database (https://www.ncbi.nlm.nih.gov/geo). GSE4290 contained 157 tumor samples and 23 normal samples. GSE12657 contained 20 tumor samples and 5 normal samples.

The original datasets were reprocessed according to certain rules. The exclusion criteria were as follows: (1) GBM samples were excluded from the GEO dataset to eliminate potential effects; (2) patients with no follow-up data were removed from the TCGA-LGG and CGGA datasets; and (3) patients with overall survival (OS) of less than 90 days in the TCGA-LGG and CGGA datasets were removed because their cause of death was most likely tumor independent. Based on this, the model was further developed and analyzed. We downloaded a gene list identified to be involved in the process of immune activity from the Immunology Database and Analysis Portal (ImmPort) database [15] (https://immport.niaid.nih.gov).

### 2.2. Differential Expression Analysis

Using the data of gene expression in GSE4290 and GSE12657, we utilized the “limma” [16] package in R software for differential expression analysis to identify differentially expressed genes (DEGs). The selection criteria were log_2_ | fold change (FC) | >1.5 and adjusted *P* values < 0.05 [17]. The “ggplot2” and “ggrepel” packages in R were used to visualize the DEGs by plotting volcanoes.

### 2.3. Functional Analysis of DEGs

To analyze the biological function of potential immune differential genes in LGGs, DEGs were included in the functional enrichment analysis as long as they were present in ImmPort's gene list. We performed Gene Ontology (GO) and the Kyoto Encyclopedia of Genes and Genomes (KEGG) analyses to look for potential biological processes and enriched pathways utilizing the cluster profiler R package. An analysis of GO terms in the categories of cellular components (CC), biological processes (BP), and molecular function (MF) was conducted. KEGG is a database integrating genomic, chemical, and systematic functional information. Adjusted *P* values < 0.05 were considered statistically significant.

ImmPort gene lists were intersected with GSE4290-LGG DEGs and GSE12657-LGG DEGs to obtain differentially expressed immune genes (DEIGs). The Spearman correlation was utilized to analyze correlations between gene expression and visualized with the “ggplot2” package. Meanwhile, the protein-protein interaction (PPI) network was retrieved from the STRING (https://string-db.org/) network and reconstructed with Cytoscape 3.7.0 software. Nodes with interaction scores greater than medium confidence (0.40) were included in the analysis.

### 2.4. Establishment of an Immune Risk Score Signature (IRSS)

The lead absolute shrinkage and selection operator (LASSO) regression analysis was performed using the DEIGs to obtain independent prognostic genes [18]. A tenfold cross validation was adopted. LASSO regression improved model accuracy and interpretability. Finally, the IRSS was constructed using LASSO regression coefficients, calculating a risk score for each patient. The formula was as follows:

(1) $$\begin{array}{c} \text{IRSS}=\overset{n}{\underset{n=1}{\sum }}\left(\text{Expression}*\text{LASSO Coefficient}\right), \end{array}$$

where Expression is the transformed relative expression value of each selected gene and LASSO Coefficient means represents the regression coefficient.

An IRSS was calculated for each TCGA-LGG sample, which was subdivided into low- and high-risk groups using the median as the cutoff. Kaplan-Meier (KM) survival plots and log-rank tests were used to show the prognostic value. The time compliance receiver-operating characteristic (ROC) curve was drawn, and the area under the curve (AUC) was computed to analyze the predictive value of IRSS for the prognosis of LGGs patients. The greater the value of AUC, the greater the consistency between predicted survival and actual survival. A decision curve analysis (DCA) was calculated at 3 years to evaluate the clinical usefulness of the constructed prognostic model [19]. The *x*-axis represents the probability threshold, or the threshold probability, and the *y*-axis represents the net benefit.

### 2.5. Immune Infiltration Analysis of Model Genes

A gene set variation analysis (GSVA) score of 6 gene integration was selected to analyze immune infiltration in TCGA samples using the Gene Set Caner Analysis (GSCA) (http://bioinfo.life.hust.edu.cn/GSCA) online tool. The GSVA score represents, in an unsupervised case, changes in gene set activity within a given cancer sample population, calculated by the R package GSVA. Briefly, the GSVA score represents a comprehensive level of gene set expression with a positive correlation with gene expression. The association between immune cell infiltration and genomic expression levels was expressed as a correlation coefficient, which was assessed by Spearman's correlation analysis. *P* values were adjusted for the false discovery rate (FDR). FDR < 0.05 was examined as statistically significant.

### 2.6. Construction of Nomogram Models

Univariate Cox regression was employed to analyze the correlation between IRSS and OS, and multivariate Cox regression analysis was employed to evaluate whether the established IRSS could serve as an independent prognostic predictor. Then, we constructed a nomogram containing IRSS and different clinicopathological information using the “RMS” package. For graphical evaluation, calibration curves at 1, 3, and 5 years were plotted, and the 45-degree line represented the best predicted value. In addition, the predictive accuracy of the nomogram was evaluated with the concordance index (C-index). Similarly, DCA at 3 years was calculated to evaluate the clinical predictive value of the nomogram.

### 2.7. External Cohort Validation

To assess the robustness and prognostic performance of the IRSS model, we selected two CGGA datasets to further validate the established model. The risk score for each patient in the CGGA cohort was calculated according to the IRSS formula established from the TCGA-LGG dataset. Multivariate Cox regression analysis combined with clinical information was carried out to understand the role of IRSS in the training set. CGGA (mRNAseq_325) and CGGA (mRNAseq_693) samples were stratified into high-risk and low-risk groups with reference to the median value of IRSS in the cohort. KM curves were used to compare the survival differences between the two groups of patients, and ROC curves were used to evaluate the accuracy of feature prediction. Likewise, DCA was employed to compare the clinical utility of both IRSS and IDH status. Meanwhile, to eliminate the potential influence of GBM samples, both cohorts were analyzed and evaluated again after excluding GBM samples.

### 2.8. Statistical Analysis

The downloaded data was organized using Excel software. An unpaired *t*-test was used to evaluate the difference in gene expression. Data analysis and visualization were done mainly by R (v3.6.1). Differences in clinical characteristics were analyzed with SPSS (version 20). Survival ROC curves were plotted using the “survival ROC” package. The KM curves were plotted by the “survival” package in R. Multivariate Cox regression analysis was performed using SPSS 20.0 with a *P* value threshold of 0.1 for inclusion in the multivariate analysis. All statistical tests were bilateral, and a *P* value < 0.05 was considered statistically significant.

## 3. Results

### 3.1. Differential Gene Analysis

**Figure 1:**
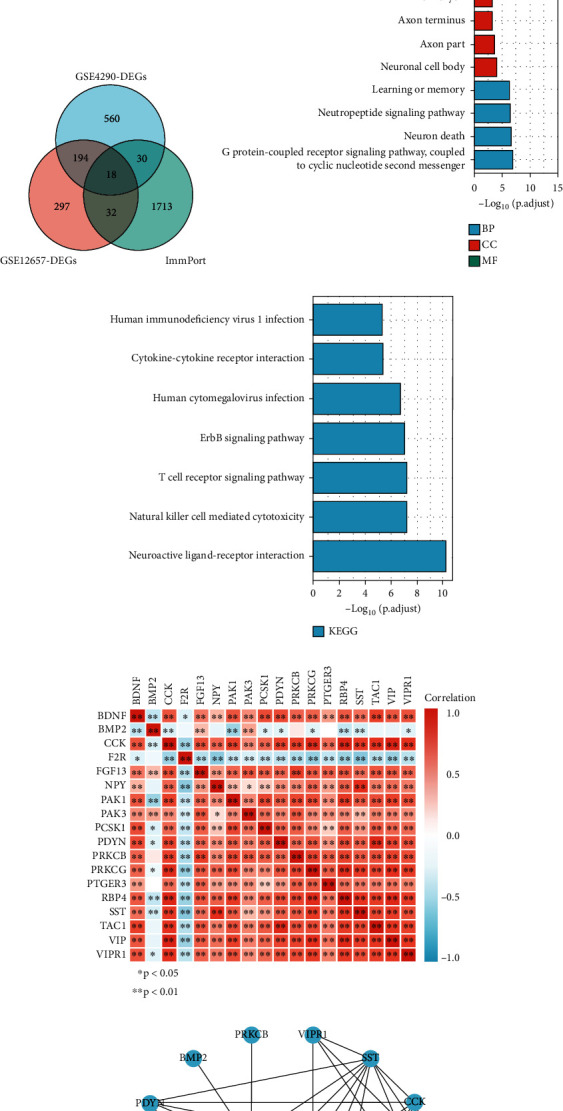
Gene expression differential analysis and potential functional analysis. (a) Volcano plot of GSE4290 differential expression. (b) Volcano plot of GSE12657 differential expression. (c) Venn diagram of DEGs. (d, e) GO and KEGG analysis of 80 immune differential genes. (f) Correlation heatmap of the intersection 18 immune differential genes. (g) Protein interaction network diagram.

**Figure 2:**
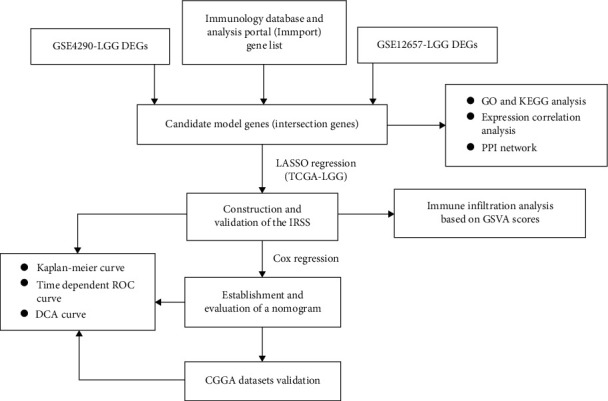
Flow chart.

The basic clinical characteristics of the reprocessed dataset are shown in Table 1. There were 76 LGGs samples in reprocessed GSE4290 and 13 LGGs samples in reprocessed GSE12657. We performed differential analysis on GSE4290 and GSE12657 to obtain genes significantly associated with LGGs. 802 DEGs were identified in GSE4290, and 541 DEGs were identified in GSE12657, and both were visualized with volcano plots (Figures 1(a) and 1(b)). The Venn diagram (Figure 1(c), supplementary Table S1) showed that 18 DEIGs were chosen from the overlap of GSE-4290, GSE-12657, and immune genes. The flow chart of this study is shown in Figure 2.

### 3.2. Functional Analysis of Immune-Related Genes

To explore the potential link between gene expression and immunity in DEGs, we performed GO and KEGG enrichment pathway analyses, and a total of 80 genes were included. GO analysis revealed significant increases in BP in G protein-coupled receptor signaling pathway, neuron death, and in response to neuropeptide signaling pathway; MF is mainly enriched in receptor ligand activity, neuropeptide hormone activity, and G protein-coupled receptor binding; CC is mainly enriched in genes regulating neuronal cell body, axon terminus, and perikaryon (Figure 1(d)). KEGG was mainly enriched in neuroactive ligand-receptor interactions, natural killer (NK) cell mediated cytotoxicity, T cell receptor signaling pathways, ErbB signaling pathways, and human cytomegalovirus infection (Figure 1(e)).

Correlation analysis showed a negative correlation between the expression of F2R, BMP2, and other genes. There was a positive correlation between each of the remaining genes (Figure 1(f)). The protein interaction network showed that somatostatin (SST) protein was the most widely connected protein (Figure 1(g)).

### 3.3. Development and Evaluation of IRSS

**Figure 3:**
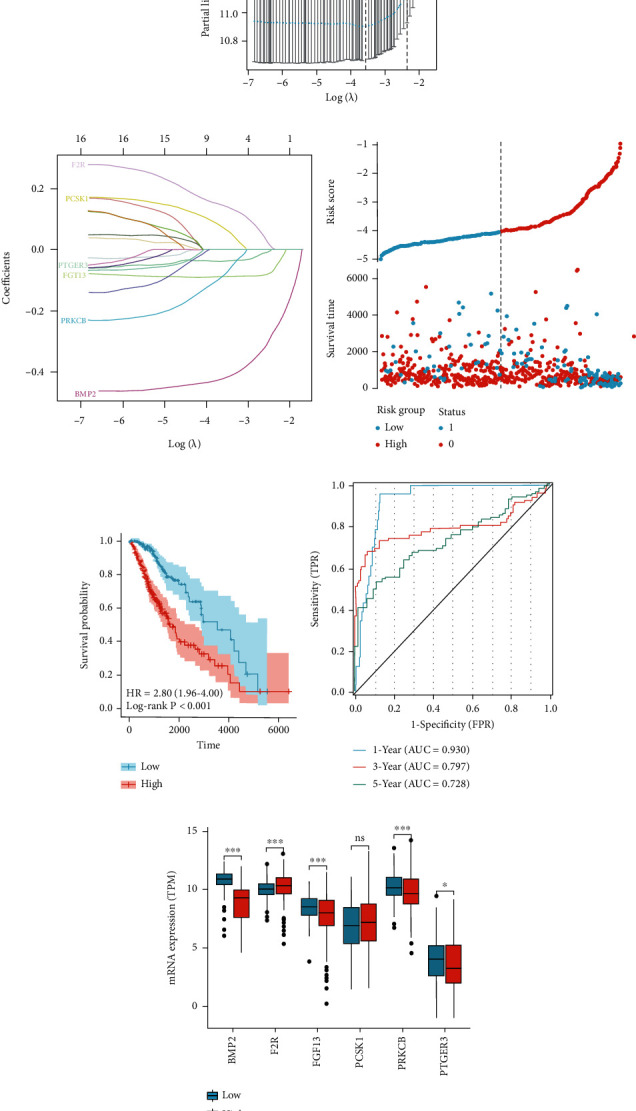
Establishment of IRSS. (a) Fine-tuning parameter selection in the LASSO model for tenfold cross validation. (b) Distribution of LASSO coefficients. (c) Risk scores and survival status of six immune genes in patients with LGGs. (d) Kaplan-Meier curves showed a significant difference in OS between the high-risk and low-risk groups in TCGA-LGG. (e) The signature was visualized by time-dependent ROC curves for predicting 1-, 3-, and 5-year survival. (f) Differential expression histogram of six immune genes between different groups. ns, *P* ≥ 0.05; ^∗^, *P* < 0.05; ^∗∗^, *P* < 0.01; ^∗∗∗^, *P* < 0.001.

To explore the possibility of immune genes as biomarkers for LGG prognosis, we selected prognostic genes from the DEIGs for further analysis. Subsequently, LASSO regression analysis obtained 6 model genes. As shown in Figures 3(a) and 3(b), the model was the best fit with a penalty coefficient of 6, and the corresponding six immune genes were BMP2, F2R, FGF13, PCSK1, PRKCB, and PTGER3 (Figure 3(e)). LASSO regression analysis of the six immune genes entered into the model yielded corresponding regression coefficients of -0.415, 0.142, -0.088, 0.068, -0.078, and -0.036 for each gene. According to the above formula, IRSS was finally established: IRSS = (−0.415 × expressionBMP2) + (0.142 × expressionF2R) + (−0.088 × expressionFGF13) + (0.068 × expressionPCSK1) + (−0.078 × expressionPRKCB) + (−0.036 × expressionPTGER3).

Following the above formula, we calculated a risk score for each patient in the study cohort. The sample was then divided into a high-risk group and a low-risk group according to the median IRSS. The KM curve results showed that the high-risk group had a worse prognosis than the low-risk group (Figure 3(d), log − rank *P* < 0.001; HR = 2.80, 95%CI = 1.96 − 4.00). The ROC curve was used to assess the accuracy of the developed prediction model for OS in patients with LGGs. As shown in Figure 3(e), the AUC values at 1, 3, and 5 years were 0.930, 0.797, and 0.728, respectively, which illustrated the robustness and accuracy of the model in predicting the prognosis of patients. There was no significant difference in the expression of the PCSK1 gene between the two groups (Figure 3(f)).

### 3.4. Immune Infiltration of Model Genes

Immune cell infiltration is a significant factor in the tumor microenvironment. Based on GSVA scores, immune infiltration analysis revealed significant negative correlations between model gene set expression and multiple immune cells, including mainly dendritic cells (DC), macrophages, helper T cell (Th) 1, Th 2, and monocytes. In addition, a negative total immune infiltration score indicated that this gene set was linked to immunosuppression (Table 2).

### 3.5. Construction of a Nomogram Incorporating Clinical Features

**Figure 4:**
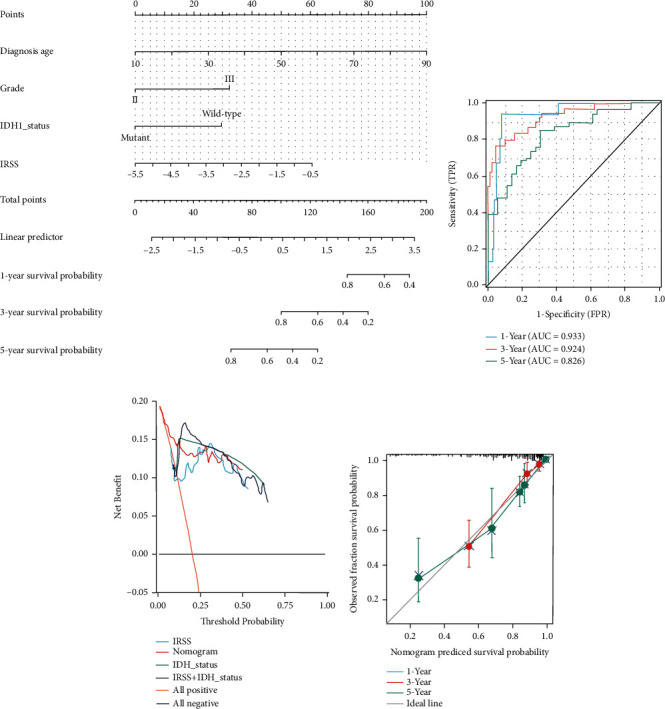
Nomogram establishment and assessment. (a) Nomogram for predicting OS in patients with LGGs. (b) Time-dependent ROC curve. (c) Decision curve analysis to assess 3-year IRSS, IDH status, and nomogram net benefit. (d) The calibration curve for predicting OS rate at 1, 3, and 5 years.

To determine the prognostic value of the established IRSS, we performed univariate and multivariate Cox regression analyses in combination with common clinicopathological features. The risk score, tumor grade, IDH status, and age were prognostic predictors in TCGA-LGG patients, with the exception of telomerase reverse transcriptase (TERT) promoter status, TP53 status, gender, and 1p/19q status. Besides that, the risk score was also an independent predictor in the multivariate Cox regression analysis (Table 3). The above results proved that the established IRSS could serve as a reliable and novel prognostic biomarker. Nomograms are widely utilized in the prognostic evaluation of tumors, which quantify statistical prediction models as estimates of event probability tailored to a single patient situation. Therefore, we built a nomogram prognostic assessment map containing multiple clinicopathological features and IRSS to individualize the prognosis of patients. The score of each variable can be comprehensively calculated to predict the prognosis of LGGs patients (Figure 4(a)).  Figure 4(b) presents the ROC curves at 1, 3, and 5 years for the nomogram with AUC values of 0.933, 0.924, and 0.826, respectively. The calibration plot showed a better diagonal fit (Figure 4(d)).

### 3.6. Model Evaluation

Glioma patients' IDH status is a recognized indicator that can affect the prognosis of glioma patients. We calculated the C-index to assess accuracy. The C-indices of the nomogram, risk signature, IDH status, and IDH + risk signature were 0.828 (0.803-0.853), 0.708 (0.666-0.750), 0.749 (0.719-0.779), and 0.759 (0.722-0.796), respectively. In summary, the TCGA-LGG cohort had moderate predictive power for IRSS, but lower than traditional IDH status. Nomogram prediction accuracy, integrating multiple pieces of information, was the most robust. Consistent with the above results, the DCA plot (Figure 4(c)) also demonstrated that the nomogram had better clinical application value.

### 3.7. External Validation

**Figure 5:**
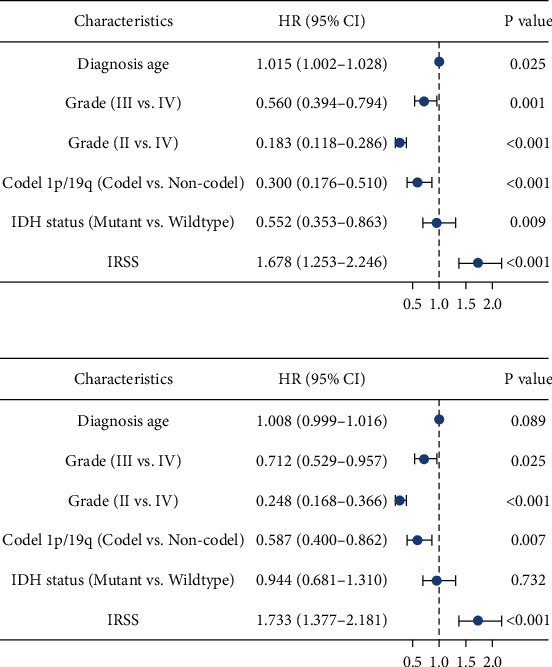
Forest plot of multivariate Cox regression analysis in the training set. (a) CGGA (mRNAseq_325); (b) CGGA (mRNAseq_693).

Two CGGA cohort datasets with similar tissues were employed to validate the general applicability of IRSS. First, a risk score was calculated for each patient in the CGGA cohort based on the IRSS formula that had been obtained. Multivariate Cox regression analyses in the test set confirmed that IRSS was also an independent predictor (Figure 5). In addition, the samples were separated into high- and low-risk groups according to the median IRSS of each cohort.

**Figure 6:**
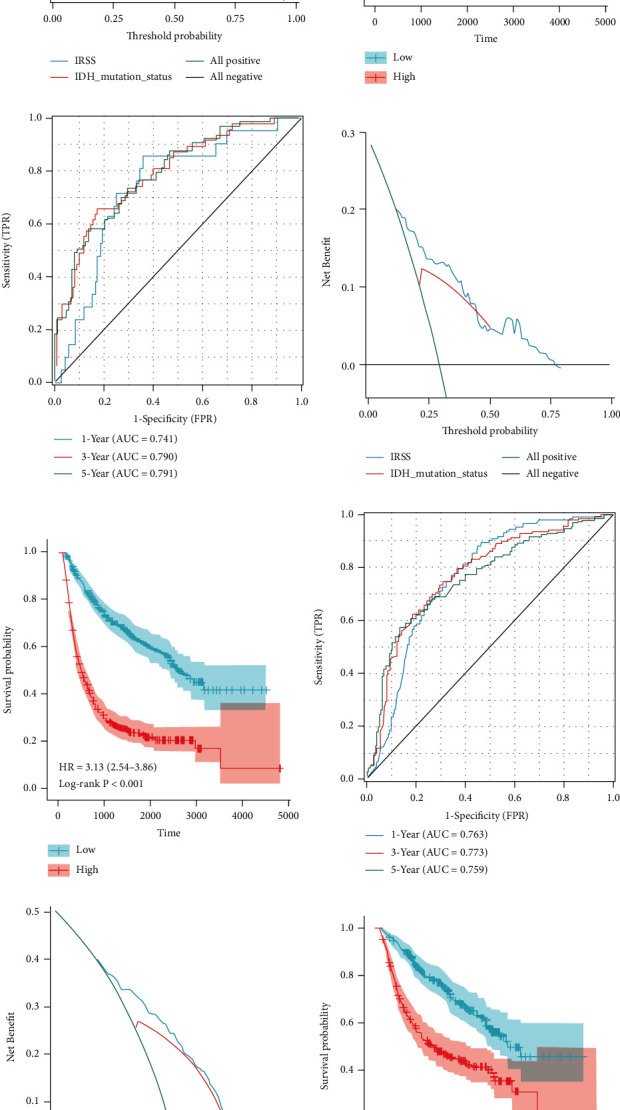
Validation in the CGGA cohort included Kaplan-Meier curves, time-dependent ROC curves, and decision curves. (a–c) CGGA (mRNAseq_325); (d, e) CGGA-LGG (mRNAseq_325); (g–i) CGGA (mRNAseq_693); (f–l) CGGA-LGG (mRNAseq_693).

The results of KM analysis confirmed that the low-risk group had a significantly better prognosis than the high-risk group in the four cohorts (Figures 6(a), 6(d), 6(g), and 6(j)), consistent with the results of TCGA-LGG. The areas under the time-dependent ROC curves of the four cohorts all showed good agreement between model's predicted OS and the actual OS (Figures 6(b), 6(e), 6(h), and 6(k)). The C-index and DCA results showed that the prognostic predictive ability of IRSS for LGG was superior to that of IDH status (Figure 6(c), 0.705 vs. 0.630; Figure 6(f), 0.708 vs. 0.617; Figure 6(i), 0.711 vs. 0.655; Figure 6(l), 0.676 vs. 0.586). In summary, we found that IRSS had broad applicability and could be used as a prognostic predictive biomarker for LGGs.

## 4. Discussion

Glioma remains one of world's deadliest brain malignancies, and its prognosis varies widely [2]. Several studies have found that the process of glioma development is closely related to the immune inflammatory response [20, 21]. Meanwhile, glioma immunotherapy has been explored to varying degrees in recent years [7]. Despite the increasing number of biomarkers associated with the survival of glioma patients with the development of high-throughput sequencing technology, the regulatory mechanisms of key genes in the tumor immune microenvironment have not yet been elucidated [5, 22–24]. Therefore, our goal is to establish immune-related biomarkers to effectively predict the prognosis of glioma.

In this study, we selected the TCGA-LGG database as the training set for the feature analysis associated with tumor immune genes. First, functional enrichment analysis of DEGs showed that immune regulatory signaling pathways were significantly enriched. Intercellular signaling is an important basis for immune regulation. Tumor-induced dysfunction of T and NK cells is a way for tumor cells to evade detection and destruction by the immune system [22, 25, 26]. The PPI network map constructed revealed that the most adjacent nodes are SST. SST is a peptide hormone involved in inhibiting cell proliferation and promoting apoptosis by binding to specific cell surface SSTRs [27]. SST peptides are thought to be key physiological regulators of immune cell function [28, 29]. It has been shown that SSTs are involved in T cell proliferation and thymocyte selection [30]. Furthermore, immune infiltration indicated an association with a partial immune cell reduction. Accumulating evidence suggests that the loss of innate immune cells such as DC, macrophages, and monocytes promotes the development of a tumor immunosuppressive state as well as immune escape. [22, 31]. Thus, the set of gene signatures we identified was strongly associated with immune function.

In addition, we combined multiple clinical datasets to establish a nomogram survival probability score. Results from DCA and C-index showed that IRSS has good predictive accuracy and clinical application with considerable predictive accuracy. In glioma, IDH mutation is a good independent prognostic marker [10]. Interestingly, IRSS was less predictive than conventional IDH status in TCGA-LGG, but the results were reversed in the four cohorts of CGGA. The number of patients with IDH status differed significantly between cohorts, which may account for the different results. In any case, for the results of this study, the predictive prognostic value of IRSS was comparable to that of IDH status.

Six key prognostic immune genes have complex biological functions. Bone morphogenetic protein (BMP) is a member of the transforming growth factor-*β* (TGF-*β*) family. BMP ligands and receptors regulate multiple functions of neural stem cells throughout neural development [32]. BMP2 induces differentiation and apoptosis in tumor cells [32–34]. Fibroblast growth factor 13 (FGF13) is a member of the FGF subfamily. Some studies have reported that FGF13 regulates glioma cell invasion and bevacizumab-induced glioma invasion [35]. Coagulation factor II thrombin receptor (F2R) is a ligand of thrombin, and thrombin has an important role in tumorigenesis and development [36]. Studies show that F2R can influence platelet mobilization as well as epidermal growth factor receptor signaling pathways to promote cancer progression [37]. Proprotein convertase subtilisin/kexin type 1 (PCSK1) encodes proprotein convertase 1/3 involved in processing neuropeptide precursors in the neuroendocrine system. However, several PC1/3 knock-out mouse models have demonstrated that neuropeptides such as enkephalin participate in immune response regulation while modulating macrophage activity [38]. Prostaglandin E2 (PGE2) is a major metabolite of cyclooxygenase-2 (COX-2) in the tumor microenvironment, which is an important mediator of immune regulation [39]. The protein encoded by prostaglandin E receptor 3 (PTGER3), EP3, is one of four receptors for PGE2, which exerts multiple effects through G protein-coupled receptors as well as downstream components of cell proliferation pathways [36, 40]. Protein kinase C beta (PRKCB) is a member of the serine and threonine-specific protein kinase family. This protein kinase is involved in many different cellular functions, such as B-cell activation, apoptosis induction, and regulation of neuronal function [41].

Potential biomarkers for predicting prognosis are now widely used in glioma. Previous studies have focused on different single prognostic genes, and therefore, the findings are not robust and comprehensive. Several immune-related gene signatures have been reported, which may imply that the prognosis of gliomas is closely related to the degree of immune infiltration [9, 12, 22]. However, most of the genetic marker studies for the prognosis of LGGs patients lack external large-scale model validation. Although we used a bioinformatics approach to identify immune prognosis-related genes, this approach has limitations in clinical practice and lacks corresponding functional experiments to validate our characterization. All cohorts, although with the same mRNA sequencing data, differed in their sequencing platforms. Therefore, the model needs to be further validated in a large sample of clinical studies with uniform criteria.

## 5. Conclusion

In summary, this study elaborated a prognostic evaluation model related to immune genes. The IRSS has advantages compared to previous signatures, but its limitations cannot be ignored. These risk scores allow for a more accurate classification of LGGs patients at the molecular subtype level. Furthermore, this knowledge has the potential to be translated into meaningful practice.

## Figures and Tables

**Table 1:** The clinical characteristics of included patients in TCGA and CGGA.

Characteristic	TCGA-LGG	CGGA (mRNAseq_325)	CGGA (mRNAseq_693)	*P*
*n*	461	302	636	
Gender, *n* (%)				0.254
Female	186 (13.7%)	113 (8.3%)	270 (19.9%)	
Male	236 (17.4%)	189 (13.9%)	365 (26.9%)	
Grade, *n* (%)				<0.001
II	199 (14.7%)	97 (7.2%)	171 (12.6%)	
III	223 (16.4%)	72 (5.3%)	243 (17.9%)	
IV	0 (0%)	129 (9.5%)	222 (16.4%)	
IDH status, *n* (%)				<0.001
Mutant	196 (17.3%)	162 (14.3%)	328 (28.9%)	
Wildtype	48 (4.2%)	139 (12.3%)	260 (22.9%)	
Codel 1p/19q, *n* (%)				0.121
Codel	71 (6.4%)	62 (5.6%)	137 (12.3%)	
Noncodel	177 (15.9%)	232 (20.8%)	435 (39%)	
Diagnosis age, median (IQR)	41 (33, 53)	42 (36, 51)	43 (34, 51)	0.696

*IDH*: isocitrate dehydrogenase; *IQR*: interquartile range; *TCGA*: The Cancer Genome Atlas; *CGGA*: Chinese Glioma Genome Atlas.

**Table 2:** The correlation between immune infiltration and gene set expression (GSVA) score.

Cell type	Estimate	FDR
CD8 naive	0.242601948	6.56307*E*-08
Cytotoxic	-0.123955504	0.038948698
Tr1	-0.124189758	0.038948698
Th1	-0.273846784	4.228*E*-10
Th2	-0.279453379	1.6547*E*-10
Th17	0.183505459	0.000134952
Tfh	-0.22634499	6.40253*E*-07
Central memory	0.234462786	2.10196*E*-07
Effector memory	-0.21962321	1.53777*E*-06
DC	-0.384131451	2.11922*E*-20
Monocyte	-0.356591092	1.97859*E*-17
Macrophage	-0.407711973	3.54139*E*-23
Infiltration score	-0.425173149	2.22905*E*-25

*DC* dendritic cells, *FDR* false discovery rate.

**Table 3:** Univariate or multivariate Cox regression analysis of clinicopathological features of LGGs associated with OS.

Characteristics	Total (*N*)	Univariate analysis			Multivariate analysis	
		Hazard ratio (95% CI)	*P* value		Hazard ratio (95% CI)	*P* value
Diagnosis age	461					
≧50	145	Reference				
<50	316	0.320 (0.220-0.465)	<0.001		0.407 (0.239-0.695)	<0.001
Gender	422					
Female	186	Reference				
Male	236	1.134 (0.769-1.673)	0.525			
Grade	422					
II	199	Reference				
III	223	3.323 (2.163-5.103)	<0.001		2.567 (1.410-4.672)	0.002
IDH1 status	248					
Mutant	189	Reference				
Wild-type	59	4.927 (3.009-8.067)	<0.001		2.465 (1.238-4.907)	0.010
IDH2 status	286					
Wild-type	274	Reference				
Mutant	12	1.121 (0.351-3.579)	0.847			
Codel 1p/19q	285					
False	200	Reference				
True	85	1.249 (0.755-2.067)	0.386			
TERT promoter status	284					
Wild-type	156	Reference				
Mutant	128	0.953 (0.586-1.551)	0.848			
TP53 status	286					
Mutant	146	Reference				
Wild-type	140	0.818 (0.505-1.324)	0.414			
IRSS	461	3.172 (2.559-3.932)	<0.001		1.565 (1.052-2.329)	0.027

*CI*: confidence interval; *IDH*: isocitrate dehydrogenase; *IRSS*: immune risk score signature; *TP53*: tumor protein p53; *TERT*: telomerase reverse transcriptase.

## Data Availability

The datasets supporting the conclusions of this article are available in the public databases The Cancer Genome Atlas (TCGA, https://portal.gdc.cancer.gov/), the Chinese glioma Genome Atlas (CGGA, http://www.cgga.org.cn/), the Gene Set Caner Analysis (GSCA) (http://bioinfo.life.hust.edu.cn/GSCA), and the Gene Expression Omnibus (GEO, https://www.ncbi.nlm.nih.gov/geo/) with the accession numbers: GSE4290 and GSE12657.

## Abbreviations

AUC: Area under the curve
BP: Biological process
BMP2: Bone morphogenetic protein 2
C-index: Concordance index
CC: Cellular component
CGGA: Chinese Glioma Genome Atlas
CI: Confidence interval
COX-2: Cyclooxygenase-2
DC: Dendritic cells
DCA: Decision curve analysis
DEGs: Differentially expressed genes
DEIGs: Differentially expressed immune genes
F2R: Coagulation factor II receptor
FC: Fold change
FDR: False discovery rate
FGF13: Fibroblast growth factor 13
GBM: Glioblastoma
GEO: Gene Expression Omnibus
GSCA: Gene Set Caner Analysis
GSVA: Gene set variation analysis
GO: Gene Ontology
IDH: Isocitrate dehydrogenase
ImmPort: Immunology Database and Analysis Portal
IRSS: Immune risk score signature
KEGG: Kyoto Encyclopedia of Genes and Genomes
KM: Kaplan-Meier
LASSO: Lead absolute shrinkage and selection operator
LGGs: Lower-grade gliomas
MGMT: Methylguanine methyltransferase
NK: Natural killer
OS: Overall survival
PCSK: Proprotein convertase subtilisin/kexin
ROC: Receiver-operating characteristic
PPI: Protein-protein interactions
PRKCB: Protein kinase C beta
PTGER3: Prostaglandin E receptor 3
SST: Somatostatin
TCGA: The Cancer Genome Atlas
TGF-*β*: Transforming growth factor-*β*
Th: Helper T cell
TP53: Tumor protein p53
TERT: Telomerase reverse transcriptase
WHO: World Health Organization.

## Supplementary Materials

Supplementary Materials Supplementary Table S1: Venn diagram data.
