# Supplementary material for: A Risk Score Signature Consisting of Six Immune Genes Predicts Overall Survival in Patients with Lower-Grade Gliomas
Source: Comput Math Methods Med. 2022 Feb 11;2022:2558548. doi: 10.1155/2022/2558548 (PMC8856808; doi:10.1155/2022/2558548)
Supplement: Supplementary Materials — Supplementary Table S1: Venn diagram data. [file 2558548.f1.pdf]

Table S1. Venn diagram data

Only GSE4	Only GSE1	Only ImmP	GSE4290-D	GSE4290-D	GSE12657-	GSE4290-DEGs AND GSE12657
TOP2A	CALB2	AZGP1	CACNG3	PSMB8	ANGPTL2	PDYN
LINC01088	KALRN	B2M	SLC30A3	EGFR	CCL2	CCK
OLFM3	PRSS3	CALR	TIMP4	RXFP1	RASGRP1	SST
GJB6	CDS1	CANX	GABRA1	NMB	TNFRSF10	F2R
NDC80	SLC6A15	CD1A	DDN	CXCL14	PENK	RBP4
CES4A	PCDH8	CD1B	GABRA5	CRH	CD14	VIP
IRX3	CNKSR2	CD1C	WIF1	FABP6	KLRC3	BMP2
LFNG	SNAP25	CD1D	SV2B	MCHR2	PPP3R1	PCSK1
MARVELD	CADPS	CD1E	CPNE6	CALCRL	ISG20L2	TAC1
HES6	CAMK1G	CD4	SOSTDC1	MSTN	PTHLH	PRKCB
CPXM1	PSD	CD8A	ID3	PAK6	HMOX1	PAK1
LGI3	DCLK1	CD8B	PDE1A	PCSK2	IL11RA	BDNF
UHRF1	DGKB	CD74	SYNGR3	CHGA	PTK2B	NPY
CREG2	TGFBI	CREB1	ITPKA	SEMA4D	PPP3CB	PTGER3
MAL2	PPM1E	CTSB	RYR2	ERAP2	IL10RA	PRKCG
RAB3C	CUX2	CTSE	ANO3	HNF4G	PNOC	FGF13
LCAT	SPTBN2	CTSL	SYN2	TNC	NEDD4	PAK3
PNMAL2	GNAO1	CTSS	MTHFD2	GREM1	C3AR1	VIPR1
ANKRD34	PPFIA3	FCER1G	HTR2C	TSHR	MAP2K1	
SYNPR	NEURL1	PDIA3	HPCA	SEMA3E	TNFRSF1B	
NEUROD6	CACNB1	HFE	KCNJ4	SSTR1	MAPK1	
RP11-320H	FAM65B	HLA-A	GRM3	RORB	BMP7	
GABRB2	MOXD1	HLA-B	RGS4	FABP3	CDK4	
TRHDE	PCP4	HLA-C	NELL1	IL12RB2	TNFRSF21	
MELK	AAK1	HLA-DMA	PCLO	IL7	S100A9	
BCAN	UST	HLA-DMB	SOX4	MCHR1	HLA-F	
KIRREL3	ADCY2	HLA-DOA	SLC8A2	TNFRSF19	TYROBP	
AK5	BRSK2	HLA-DOB	SLC4A3	THRB	FCGRT	
GPR22	CEP170B	HLA-DPA1	SLC17A7	MDK	CBLB	
NPY1R	DLG4	HLA-DPB1	CRYM	ADRB1	PLXNB2	
GLT1D1	PIP5K1B	HLA-DQA	NEFM		CSF2RA	
OGDHL	LRRC8B	HLA-DQA	RFPL1S		GRB2	
CBLN4	MEF2C	HLA-DQB	SNCG			
TLL2	CPVL	HLA-DRA	NEFL			
FAM19A1	ECM2	HLA-DRB	GRIN2A			
PACSIN1	ATP2B2	HLA-DRB	SULT4A1			
C3orf80	HCN2	HLA-DRB	SLC12A5			
GALNTL5	DAAM1	HLA-DRB	NPTX1			
HCN1	COL4A1	HLA-E	NRGN			
GDA	AP1S1	HLA-G	GABRG2			
PBK	NKX2-2	HLA-H	ATP8A2			
C11orf87	OLFM1	MR1	ERC2			
SERTM1	PSMB9	HSPA1A	SERPINI1			
LINC00507	AQP3	HSPA1B	MICAL2			

RSPO2	FAM131A	HSPA1L	LPL
DRD1	RND2	HSPA2	RIMBP2
C1orf115	CDH13	HSPA4	WNT10B
RAPGEFL1	PTPRT	HSPA5	SYT1
SMPX	SLN	HSPA6	ICAM5
ERICH3	CAMK1	HSPA8	CAMK2B
EIF4EBP1	STXBP1	HSP90AA1	STX1A
SOX11	MAPK8IP2	HSP90AB1	ST18
PCDHGA8	GAD1	ICAM1	CAMK2A
MAP7D2	MMD	IFNA1	NPTX2
TMEM100	BAIAP2	IFNA2	GABRA2
OPALIN	CDKL5	IFNA4	STMN2
TMEM235	UCHL1	IFNA5	PHYHIP
NECAB1	RAP1GAP	IFNA6	DLG2
DLGAP3	PRKAR2B	IFNA7	VSNL1
NES	CACNB3	IFNA8	CA11
RP11-332H	PDE1B	IFNA10	KIAA0319
ATP2B3	CACNB2	IFNA13	PARM1
HHIP	FAM49A	IFNA14	NPTXR
TMEM191	WDR47	IFNA16	SNCB
NWD2	SKAP2	IFNA17	MYT1L
RTBDN	PLD3	IFNA21	TRAF3IP2
CALY	PRSS2	IFNG	RCAN2
ATOH7	DNM3	KIR2DL1	CCKBR
CHRM3	CDK5R1	KIR2DL2	MYC
MFSD4	ATP1B1	KIR2DL3	ETV1
C1QTNF4	MCM7	KIR2DL4	PPL
GLS2	TP53I3	KIR2DS1	KCNJ3
RAP1GAP2	IDS	KIR2DS3	PTPN20B
CDKL2	REV3L	KIR2DS4	GRM1
CYP4X1	MMP16	KIR2DS5	PLK2
CHRM1	EIF4E	KIR3DL1	RAB3A
SYTL5	IDH3A	KIR3DL2	CDH12
GBX2	DYNLT1	KLRC1	ASCL1
SNCA	KDM5A	KLRC2	CAP2
PTPN5	RFK	KLRD1	SH3GL3
FLJ16779	NUP98	LTA	NECAB2
DLL3	CTSO	CIITA	FAM189A1
SLC26A4-A	EFNB3	MICA	MAL
RBFOX1	PAIP1	MICB	KIAA1107
SYT13	DPY19L2P	NFYA	RIMS3
AKAP5	GAS7	NFYB	KIAA0513
RP1-212P9.	PRPS2	NFYC	TBR1
LRRC7	OPA1	LGMN	ENC1
RHBDL1	OLIG2	PSMC1	DNM1
TTC9B	SRD5A1	PSMC2	MAST3

CHST9	C1orf95	PSMC3	CRHBP
RBFOX3	CPNE1	PSMC4	KCNK1
HOOK1	BARD1	PSMC5	GLS
PPP4R4	CDK2	PSMC6	SPOCK3
JAZF1-AS1	SLC12A4	PSMD1	GRIN1
DLX1	RAB13	PSMD2	EPHA5
NKAIN2	MOAP1	PSMD3	HTR2A
EZH2	ENO2	PSMD4	KIAA1045
IRX1	NUP188	PSMD5	DOC2A
ADAMTS8	CCND1	PSMD7	SCN2A
NUDT14	TUSC3	PSMD8	BCHE
TMEM130	VSIG4	PSMD10	HSPA12A
GNG3	AK2	PSMD11	BSN
MIR7-3HG	ASS1	PSMD13	DMTN
SOWAHA	LPGAT1	PSME1	NRXN3
GPR37	LYRM9	PSME2	MDFI
S1PR5	LILRB4	RELB	MYRIP
TMEM151	C1orf216	RFX5	CLCN4
WNK2	KLK7	RFXAP	SORBS2
MYO1D	ATP6V1H	SLC10A2	STON1
CLEC4G	HMHA1	TAP1	KCNAB2
KRT222	SYNJ1	TAP2	CABP1
ASIC2	MAST1	TAPBP	NCDN
KIF4A	NEUROD2	THBS1	RAPGEF5
TMEM155	FBXL2	SEM1	PTPRR
RASAL1	NSG1	KLRC4	PRKCZ
UNC13C	SCAMP5	AP3B1	LPCAT4
KCNJ9	TP53I11	RFXANK	XK
RP11-182J	THEMIS2	PSMD6	NEFH
GPR83	ARPP19	PSME3	KCNJ6
SLC17A6	TNR	PSMD14	NELL2
ARX	ABCB9	CLEC4M	LDB2
RP11-389G	HOMER1	IFI30	NID1
MUM1L1	GPR6	PROCR	PPFIA2
MAK	MAP3K10	ADRM1	RUNDC3A
LOC28457	CA4	ECPAS	RAB40B
NAPB	PNMA2	TRPC4AP	ZNF536
KIF20A	LGALS3B	CD209	CHD5
SYT4	KIF3C	UBXN1	BCL11A
PPP2R2C	RASGRF1	ERAP1	HPRT1
RASGEF1	MAP2K4	TAPBPL	CDH18
RASGRF2	NEDD9	KIR2DL5A	LGI1
CDH8	MDC1	ULBP3	GAD2
MAML2	SHANK2	ULBP2	ZNF365
FAM19A2	ATP6V1B2	ULBP1	ADCY1
RP11-710C	NECAP1	KIR3DL3	RPH3A

SYN1	CAMTA2	RAET1E	GUCY1B3
SNCAIP	SRPX	RAET1L	PPM1H
BEX5	OXCT1	UBR1	TPPP
PEX5L	GABARAP	RAET1G	NAV3
CYP26B1	TRIOBP	PDIA2	LPPR4
ERMN	ZNF204P	HAMP	SYP
RTN4RL2	SLC7A7	PI3	DYNC1I1
TENM2	TMEM246	CAMP	AMPH
STXBP6	PPP1R13B	DEFB4A	SLC2A3
CEP55	GPX7	PPBP	FRRS1L
FERMT1	MATK	REG3G	TTC9
PPP1R16B	RTN2	CXCL16	DOCK3
ARHGAP4	ARHGEF9	SLPI	DGKZ
CRNDE	PREPL	CXCL8	RIMS2
SLC5A11	AKAP13	CXCL10	LY6H
ST6GALN	IDH1	CXCL9	SNAP91
TUNAR	DNMBP	CXCL5	CDKN2D
RNF128	CDK17	CXCL11	EPB41L1
PALM2	RAB31	CXCL6	TAGLN3
HS3ST4	LAPTM5	CXCL1	GPRASP1
ZNF385B	MSL3	CXCL12	KCNQ2
NKAIN4	RTN1	CXCL13	GOT1
SVOP	ZNF529	CXCL2	NEDD4L
KIF17	RECQL	PF4	LYPD1
HPCAL4	RUNDC3B	XCL1	YWHAH
PCDHB11	PDE4A	CXCL3	MAP3K9
SHD	KLF10	DEFB103B	NIPAL3
TTLL7	PCGF2	CCL13	EPHB6
DNAJA4	LIMK2	CCL1	APLP1
CNTN2	SLC4A7	DEFB1	ELAVL2
CELF4	SUCLA2	CCL8	PHLDA1
RP11-215H	ATP6V1D	ELANE	RGS7
HSPB3	REEP1	DEFB103A	PDE2A
NGB	VAMP2	DEFA3	ZBTB20
BEND4	ZNF804A	DEFA1	SYNGR1
HIST1H4J	TSPYL4	TMSB10	ARHGAP44
SCN3B	NDEL1	DEFA6	SYT5
ST8SIA5	MLLT11	DEFA5	SCN2B
LOC38989	CDK5	DEFA4	TCF12
ZMAT4	BTBD3	LCN2	PAK7
DGKE	PARVB	LCN1	CHN1
VSTM2L	PXDC1	COLEC10	PTPN3
PRRX1	PARK2	BPI	NNAT
SLC35F3	MPST	S100A8	SPINT2
CLCA4	BASP1	DCD	CACNA2D1
DIRAS2	C14orf2	LCN6	TUBA4A

RP11-469M	ATP6V1A	S100A12	PSD3
SAMD12	KIAA0368	HTN3	RAPGEF4
FXYD7	CLSTN3	LCN8	HNMT
MYRF	OGG1	DEFA1B	PTPRN
AF131215.4	AKR1C3	CCR10	APOC1
SLC24A2	PFKP	CELA1	PART1
LOC729732	SOX13	DEFB106A	SPARC
CTB-78F1.	NUDT1	BPIFC	PRKAR1B
CNTN4	MPP2	MMP12	ATRNL1
ZCCHC12	HLF	BPIFB6	WDR7
LOC401220	PLCH1	LEAP2	RP2
CARNS1	TSPYL2	SFTPD	SH3BP5
RIT2	UBE2V2	LCN9	
PCDHB9	TXNIP	BPIFB2	
EMX1	CAMTA1	PTGDS	
JAKMIP3	APC2	TMSB4X	
WSCD2	ACOX1	PGLYRP1	
FNDC9	PALM	ZC3HAV1	
PITPNM3	CCDC93	TMSB15A	
KLHL26	SRPK2	S100B	
CCDC80	RASSF2	S100A13	
PDE1C	TBC1D9	S100A6	
WEE1	RGL2	DEFB119	
UBE2QL1	ADAM17	DEFB107A	
JPH1	DMXL2	DEFB105A	
CTC-471C1	ACOT7	SERPIND1	
CPLX2	NPTN	DEFB129	
DLGAP2	ZNF22	DEFB127	
RAB3B	ARL6IP1	S100P	
FOXD1	FKBP8	S100A7	
CDH19	SMARCD2	DEFB104A	
JAKMIP1	SBF1	DEFB126	
TFAP2A	MAPRE3	DEFB106B	
TAS2R5	KIF5A	DEFB104B	
LOC440934	IQSEC3	DEFB107B	
KCNH3	AJAP1	PGLYRP3	
44266	LBR	PGLYRP2	
CNGA3	SLBP	S100A10	
PGM2L1	GHITM	S100A2	
RP11-116O	MAPRE2	DEFB125	
COL24A1	AP3M2	DEFB123	
CNDP1	R3HDM1	DEFB105B	
SLC6A17	KCND3	DEFB132	
THEMIS	ZCCHC24	BPIFB3	
KCTD4	MAPK9	LCN12	
GRIN2B	MCL1	PGLYRP4	

SDR16C5	NUPL1	S100A11
ITPR1	DUSP3	S100A5
KIF18A	TUBB2A	S100A3
FEZF2	STX10	S100A1
TPPP3	RNMT	DEFB128
SPSB4	GNB5	DEFB108B
BTBD17	RAMP1	HTN1
ARHGDIG	DLG5	LMBR1L
NCS1	GULP1	S100A7A
LINC00889	G3BP2	DEFB118
NEGR1	NRXN1	COLEC12
UNC5D	ANK2	TMSB4Y
WBSCR17	UCKL1	DEFB131A
VSTM2A	DLGAP1	DEFB134
CACNA1B	MMP17	DEFB130A
FOXM1	TOMM34	DEFB124
GRM2	RALGPS1	DEFB121
GSX1	GM2A	DEFB116
TSPAN12	ZNF384	DEFB115
STK17B	CDC25B	DEFB114
SLC7A14	DSTYK	DEFB113
TRIM36	NME4	DEFB112
GPR62	UNC13A	DEFB110
SYNJ2	AGAP2	TMSB15B
EFNA2	KIF5C	DEFB133
LRFN2	PHLPP2	S100Z
PRRG3	FAAH	MAVS
SELE	IGFBP4	TMSB4XP8
FOSB	YIF1A	S100A14
MEX3A	PPWD1	LCN10
TRIM59	TBC1D30	S100A16
CPNE4	RNF41	DEFB136
SMOC1	QDPR	DEFB135
BHLHE22	NAP1L3	DEFB117
ADAMTS1	MED12	ZC3HAV1L
PRR18	TLN1	S100A7L2
FCGBP	ATAD2B	MBL3P
LPAR1	MAPK10	DEFB4B
VEPH1	LZTS3	BPIFB4
RNF157-A[illegible]	NCKAP1L	IFNAR1
SEC14L5	SEC23A	AZU1
SLITRK4	RNF11	DEFB131B
CALB1	PVRL2	DEFA1A3
CACNA2D	SLC4A2	LCN1P1
MACROD[illegible]	CLN3	S100G
ABCA1	YWHAB	DEFA7P

EPHX4	ANXA11	DEFB130B
LOC28514	PRNP	DEFB108F
TSPAN11	PAPOLA	DEFB131C
DUSP2	LARGE	TCHHL1
LINC0046	PDLIM5	TINAGL1
ENPP2	MAPK6	IFNGR1
LOC38983	PTTG1IP	SLC22A17
CPEB1	CAPN2	WFIKKN1
SIX1	DLX2	WFDC2
IPCEF1	SERINC3	IL6
E2F8	SSX2IP	UMODL1
HS6ST3	TRIM2	TGFB1
SYT16	APBB1	PF4V1
NTN1	STK10	MMP9
CDKN2B	SALL2	ANOS1
AF070581	ELAVL4	TLR4
FKBP10	CDK14	SPAG11B
CCDC167	SLC27A2	A2M
GRM5	SEPT8	NFKB1
PCTP	GNAQ	APOBEC3G
ANK3	EFS	NOD2
LOC28617	NUMA1	MBL2
CLDN10	TRIM37	SFTPA1
FBXW7		RBP1
LOC100506731		TLR2
KIAA1211L		SLC40A1
EEF1A2		PLAU
ASIC4		IL1B
LINC00645		PAEP
NUAK1		HJV
C7orf57		MUC5AC
RIMS1		OBP2A
CDH9		PLTP
SLC26A4		MX1
COX7A1		DDX58
SIX4		IFNL1
TMEM132D		IRF3
PPFIA4		SFTPA2
RPRML		LPA
TUBB4A		LBP
HMGCLL1		NOX4
ZIC1		LTF
LRRC2		IFNB1
ADAMTS6		RBP5
OR2L13		FABP7
RALYL		FABP5

CLEC2L	FABP2
LINC01279	FABP4
TMEM38A	R3HDML
CSRP2	BPIFA3
RBM24	BPIFB1
LOC100129973	OASL
COL26A1	CRABP2
NCEH1	CRABP1
CYP46A1	RBP7
VANGL2	DUOX1
STPG1	OBP2B
NUSAP1	RBP2
ENTPD3	LCN15
DDIT4	CETP
SLC32A1	FABP12
GABRA4	FABP9
EPB41L4B	BPIFA1
EPB41L3	LCNL1
NSUN7	C8G
PLEKHH3	SPAG11A
STMN4	PI15
NINJ2	NOX1
BUB1	PMP2
TMEM56	APOD
AHNAK2	ORM2
DNAJC6	ORM1
HMX1	TNF
SOX6	CTSG
ASPA	PRTN3
MRAP2	PML
LINC00086	AEN
MCTP1	CYBB
BOK	BPIFA2
CLSTN2	ISG20
CHN2	BCL3
LINC00836	NOX5
TSPYL5	NOX3
FRMPD4	DUOX2
DLGAP5	TLR3
BEST3	TFRC
NT5DC2	IFIH1
CYP26A1	LRP1
PPP1R14A	TRIM5
KCNA4	IDO1
ST8SIA3	GDF15
PRO1082	ADIPOQ

ELOVL4	STAT3
LINGO2	STAT1
DOK6	IFNL2
NGEF	SOCS3
SELL	SEMG1
KCNQ5	TNFSF10
RYR3	CCL20
ABCA5	SOCS1
SLITRK1	RNASEL
RP4-593C16.3	IRF1
GALNT14	IL15
SRCIN1	APOBEC3F
C1orf192	PLAAT4
FKBP1B	CHIT1
DCTN1-AS1	CD40
P2RX5	TLR7
SYTL4	PPIA
RP3-406A7.7	ZYX
NDFIP2	NLRX1
RTN4RL1	PGC
NUF2	VEGFA
UNC5A	IKBKE
RERG	ISG15
ID4	DHX58
NPAS1	TNFAIP3
F5	TFR2
44259	FCN2
PPP1R14B	MUC4
PRKX	ELN
LHFPL3	IL27
FAM110C	MAPT
LOC101927988	LYZ
CPLX1	CCL5
LNX1	LEP
L1CAM	CYLD
REPS2	KLKB1
ADRA1B	CST4
SH2D5	CSRP1
STXBP5	MAPK14
HOXA10	JUN
BOC	ITGAV
MAG	IRF5
RAB27B	CCR6
LRTM2	IL12B
PCDHGA10	TLR8
STYK1	GNLY

FANCE	CD81
TSPAN6	EIF2AK2
LMNB1	APOM
CRISPLD1	CACYBP
CCDC3	NOD1
ACTR3C	MAPK8
FHL2	MAPK3
YJEFN3	BST2
CCDC68	BPHL
KIAA1324	PLA2G2A
KCNAB1	GRN
OPCML	NEWENTRY
PCP4L1	PDGFRA
TNIP3	GNAI1
CDK5R2	WNT5A
CLMN	FURIN
CBR1	ADAR
MCM5	TYK2
NIPAL2	NOS2
CAMK4	TRAF3
CELF5	TPT1
C1QL1	TPM2
CKAP2L	NEO1
PRKCD	AHNAK
PLLP	TLR1
CHAC1	TK2
TMEM200A	PRDX2
ZNF560	MX2
EXTL1	FGF2
LOC100506563	FGA
FAM84B	TCF7L2
SLC22A15	F2RL1
MEG3	TKFC
BDKRB2	MSR1
KIAA1598	NFKBIZ
LINC00320	LMBR1
ADAMTS19	EPPIN
PPP1R14C	SRC
TNNT1	MPO
ANKFN1	ELAVL1
SRRM4	ROBO3
PPP1R1B	SP1
NCOA7	SOD1
SULF2	PDF
FRAS1	DLL4
MSI1	ECD

KCNQ3	SLC11A1
LOC100287387	DMBT1
SV2C	STING1
FSTL5	SKIV2L
LOC100131170	SEMG2
SNX10	DES
TMEM125	DCK
CAMKV	DAXX
GALR1	TNFRSF10A
KIAA1661	EED
KCTD16	CCL4
LRP2	LIMS1
GOLGA7B	LALBA
PPEF1	APOBEC3H
NOTCH2NL	TMPRSS6
B3GALT2	SPINK5
VWA1	MARCO
LINC00301	BECN1
HMCN1	TNFSF11
KIAA1644	KNG1
CENPK	CSK
VCAN	KLRK1
STK32A	KCNH2
CRB1	JUND
CNTNAP2	JAK1
LOC100505912	CLDN4
MAFG-AS1	CCL28
STX1B	RNASE3
POFUT1	RN7SL1
ATL1	IRF7
LOC101927811	IREB2
HEY1	ILK
LOC286367	IL18
ANO4	IL17A
LRFN5	LTB4R
DOCK9	APOBEC3A
FOLH1B	MASP2
SLC39A12	TRIM27
GABRD	RELA
PCDHAC2	IL7R
NAP1L2	IL1A
MAGEE1	PTX3
SEPT4	IFNAR2
TP53	IFN1@
CCDC85A	SYTL1
TSTD1	APOBEC3C

JPH3	DDX17
UNC5C	PTGS2
ADAM11	HTR1A
RBM11	SEPTIN7
GCK	CD40LG
COL9A3	MASP1
NOMO3	PROC
NEU4	MAP2K2
CNTN3	HRG
SH3GL2	NDRG1
SOX2	IRF9
RPE65	TRIM22
SOX10	LANCL1
ANTXR1	PPP4C
SYNPO	HMGB1
SGPP2	RNASE7
ANKRD18A	ABCC4
APBA1	HGF
EGFEM1P	HDAC1
LOC100128079	IFNLR1
TYRP1	PLSCR1
GNAL	BACH2
PNMA6A	TANK
KIAA0101	PIK3CG
WDR49	ARRB1
RNF213	RSAD2
CLEC5A	STAB2
KCNIP4	TBK1
DACH2	PDGFRB
BRSK1	PDCD1
LRRC75A	ARG2
TLN2	AQP9
BFSP1	FASLG
FAM71E1	APOH
LOC285556	BIRC5
KLHL2	ANXA6
LOC100132891	IL22
GLRA2	VTN
MEGF11	VIM
DYNLT3	VCAM1
LOC285812	PRDX1
LPPR5	GFAP
PLEKHA1	GBP2
NIPAL4	ALB
ATOH8	SLC29A3
TSKU	OAS1

KCNA1	AGER
KLF8	UNC93B1
HAR1A	TNFSF4
KLK6	NOS1
SALL3	ACTG1
HS3ST2	ACTA1
DLL1	ACO1
EPHA4	SERPINA3
DTNB	CXCR1
KLF16	CCL15
	CCL14
	CCL16
	CCL19
	CCL18
	CCL17
	CCL26
	CCL22
	CCR3
	CCL4L1
	ACKR2
	CCR7
	CCL27
	CCR8
	ACKR4
	CCL21
	CCL7
	CCL3
	CCL11
	CCR5
	CCL23
	CCL25
	CCL3L3
	CCL4L2
	CCL3L1
	CCR1
	CCL24
	XCL2
	CXCR4
	CXCR6
	CCR4
	TAFA5
	TAFA3
	TAFA4
	TAFA1
	TAFA2
	CCL15-CCL14

IL4
CDH1
LTBP1
IL13
IL10
IL2
PPARG
FGR
MIF
CRP
JAK2
PTK2
PTGDR
CD86
HCK
VDR
OLR1
GRK2
TXK
RNASE2
CD79A
CD79B
LYN
SYK
BTK
BLNK
VAV3
VAV1
VAV2
RAC1
RAC2
RAC3
PPP3CA
PPP3CC
CHP1
PPP3R2
CHP2
NFAT5
NFATC1
NFATC2
NFATC3
NFATC4
HRAS
KRAS
NRAS
FOS

CARD11
BCL10
MALT1
CHUK
IKBKB
IKBKG
NFKBIA
NFKBIB
NFKBIE
CD19
CR2
PIK3R5
PIK3R1
PIK3R2
PIK3R3
PIK3CA
PIK3CB
PIK3CD
AKT3
AKT1
AKT2
GSK3B
INPP5D
CD22
CD72
PTPN6
LILRB3
FCGR2B
RASGRP3
PLCG2
IFITM1
IGH
IGHA1
IGHA2
IGHD
IGHD1-1
IGHD1-14
IGHD1-20
IGHD1-26
IGHD1-7
IGHD2-15
IGHD2-2
IGHD2-21
IGHD2-8
IGHD3-10
IGHD3-16

IGHD3-22
IGHD3-3
IGHD3-9
IGHD4-11
IGHD4-17
IGHD4-23
IGHD4-4
IGHD5-12
IGHD5-18
IGHD5-24
IGHD5-5
IGHD6-13
IGHD6-19
IGHD6-25
IGHD6-6
IGHD7-27
IGHE
IGHG1
IGHG2
IGHG3
IGHG4
IGHJ1
IGHJ2
IGHJ3
IGHJ4
IGHJ5
IGHJ6
IGHM
IGHV1-18
IGHV1-2
IGHV1-24
IGHV1-3
IGHV1-45
IGHV1-46
IGHV1-58
IGHV1-69
IGHV1-8
IGHV1-38-4
IGHV1-69-2
IGHV2-26
IGHV2-5
IGHV2-70
IGHV3-11
IGHV3-13
IGHV3-15
IGHV3-16

IGHV3-20
IGHV3-21
IGHV3-23
IGHV3-30
IGHV3-30-3
IGHV3-30-5
IGHV3-33
IGHV3-35
IGHV3-38
IGHV3-43
IGHV3-48
IGHV3-49
IGHV3-53
IGHV3-64
IGHV3-66
IGHV3-7
IGHV3-72
IGHV3-73
IGHV3-74
IGHV3-9
IGHV3-38-3
IGHV3-69-1
IGHV4-28
IGHV4-30-1
IGHV4-30-2
IGHV4-30-4
IGHV4-31
IGHV4-34
IGHV4-39
IGHV4-4
IGHV4-59
IGHV4-61
IGHV4-38-2
IGHV5-51
IGHV5-10-1
IGHV6-1
IGHV7-4-1
IGHV7-81
IGK
IGKC
IGKDEL
IGKJ
IGKJ1
IGKJ2
IGKJ3
IGKJ4

IGKJ5
IGKV@
IGKV1-12
IGKV1-13
IGKV1-16
IGKV1-17
IGKV1-27
IGKV1-33
IGKV1-37
IGKV1-39
IGKV1-5
IGKV1-6
IGKV1-8
IGKV1-9
IGKV1D-12
IGKV1D-13
IGKV1D-16
IGKV1D-17
IGKV1D-33
IGKV1D-37
IGKV1D-39
IGKV1D-42
IGKV1D-43
IGKV1D-8
IGKV2-24
IGKV2-28
IGKV2-30
IGKV2-40
IGKV2D-24
IGKV2D-28
IGKV2D-29
IGKV2D-30
IGKV2D-40
IGKV3-11
IGKV3-15
IGKV3-20
IGKV3-7
IGKV3D-11
IGKV3D-15
IGKV3D-20
IGKV3D-7
IGKV4-1
IGKV5-2
IGKV6-21
IGKV6D-21
IGKV6D-41

IGL
IGLC1
IGLC2
IGLC3
IGLC6
IGLC7
IGLJ
IGLJ1
IGLJ2
IGLJ3
IGLJ4
IGLJ5
IGLJ6
IGLJ7
IGLV@
IGLV1-36
IGLV1-40
IGLV1-44
IGLV1-47
IGLV1-50
IGLV1-51
IGLV10-54
IGLV11-55
IGLV2-11
IGLV2-14
IGLV2-18
IGLV2-23
IGLV2-33
IGLV2-8
IGLV3-1
IGLV3-10
IGLV3-12
IGLV3-16
IGLV3-19
IGLV3-21
IGLV3-22
IGLV3-25
IGLV3-27
IGLV3-32
IGLV3-9
IGLV4-3
IGLV4-60
IGLV4-69
IGLV5-37
IGLV5-39
IGLV5-45

IGLV5-48
IGLV5-52
IGLV6-57
IGLV7-43
IGLV7-46
IGLV8-61
IGLV9-49
C3
C5
CCL3P1
CKLF
CMA1
CX3CL1
CXCL17
CCN1
EDN1
EDN2
EDN3
FGF10
LECT2
PPBPP1
PROK2
SAA1
SAA2
SBDS
SEMA3A
SEMA3B
SEMA3C
SEMA3D
SEMA3F
SEMA3G
SEMA4A
SEMA4B
SEMA4C
SEMA4F
SEMA4G
SEMA5A
SEMA5B
SEMA6A
SEMA6B
SEMA6C
SEMA6D
SEMA7A
SLIT1
SLIT2
TYMP

C5AR1
CCR9
CCRL2
CMKLR1
CX3CR1
CXCR3
CXCR5
ACKR3
CYSLTR1
CYSLTR2
ACKR1
EDNRA
EDNRB
FPR1
FPR2
GPR17
GPR32
GPR33
PTGDR2
C5AR2
CXCR2
LTB4R2
PLAUR
PLXNA1
PLXNA2
PLXNA3
PLXNA4
PLXNB1
PLXNB3
PLXNC1
PLXND1
PTAFR
ROBO1
ROBO2
RXFP3
XCR1
ADM
ADM2
AGRP
AGT
AMBN
AMELX
AMH
ANGPTL5
ANGPTL7
APLN

AREG
MANF
CDNF
ARTN
AVP
BMP1
BMP10
BMP15
BMP3
BMP4
BMP5
BMP6
BMP8A
BMP8B
BTC
MYDGF
CALCA
CALCB
CAT
CD320
CD70
ADA2
CER1
CGA
CGB3
CGB1
CGB2
CGB5
CGB7
CGB8
CHGB
CLCF1
CLEC11A
CMTM1
CMTM2
CMTM3
CMTM4
CMTM5
CMTM6
CMTM7
CMTM8
CNTF
CORT
CSF1
CSF2
CSF3

CSH1
CSH2
CSHL1
CSPG5
CTF1
CCN2
DKK1
EBI3
EGF
EPGN
EPO
EREG
ESM1
FAM3B
FAM3C
FAM3D
FGF1
FGF11
FGF12
FGF14
FGF16
FGF17
FGF18
FGF19
FGF20
FGF21
FGF22
FGF23
FGF3
FGF4
FGF5
FGF6
FGF7
FGF8
FGF9
VEGFD
FIGNL2
FLT3LG
FSHB
GAL
GALP
GAST
GCG
GDF1
GDF10
GDF11

GDF2
GDF3
GDF5
GDF6
GDF7
GDF9
GDNF
GH1
GH2
GHRH
GHRL
GIP
GKN1
GMFB
GMFG
GNRH1
GNRH2
GPHA2
GPHB5
GPI
GREM2
GRP
GUCA2A
HBEGF
HDGF
HDGFL3
IAPP
IFNE
IFNK
IFNW1
IGF1
IGF2
IL11
IL12A
IL16
IL17B
IL17C
IL17D
IL17F
IL19
IL1F10
IL36RN
IL36A
IL37
IL36B
IL36G

IL1RN
IL20
IL21
IL23A
IL24
IL25
IL26
IFNL3
IL3
IL31
IL32
IL33
IL34
IL5
IL6ST
IL9
INHA
INHBA
INHBB
INHBC
INHBE
INS
INS-IGF2
INSL3
INSL4
INSL5
INSL6
JAG1
JAG2
FGF7P6
FGF7P3
KITLG
KL
LACRT
LEFTY1
LEFTY2
LHB
LIF
LRSAM1
LTB
LTBP2
LTBP3
LTBP4
MIA
MLN
NAMPT

NDP
NENF
NGF
NODAL
CCN3
NPFF
NPPA
NPPB
NPPC
NRG1
NRG2
NRG3
NRG4
NRTN
NTF3
NTF4
NTS
NUDT6
OGN
OSGIN1
OSM
OSTN
OXT
ENDOU
PDGFA
PDGFB
PDGFC
PDGFD
PDGFRL
PGF
PMCH
POMC
PPBPP2
PPY
PRL
PRLH
PROK1
PSPN
PTH
PTH2
PTN
PYY
QRFP
RABEP1
RABEP2
REG1A

RETN
RETNLB
RLN1
RLN2
RLN3
SCG2
SCGB3A1
SCT
AIMP1
SECTM1
SLURP1
SPP1
STC1
STC2
TDGF1
TDGF1P3
TG
TGFA
TGFB2
TGFB3
THPO
TNFRSF11B
TNFSF12
TNFSF13
TNFSF13B
TNFSF14
TNFSF15
TNFSF18
TNFSF8
TNFSF9
TOR2A
TRH
TSHB
TSLP
TXLNA
UCN
UCN2
UCN3
UTS2
UTS2B
VEGFB
VEGFC
VGF
ACVR1B
ACVR1C
ACVR2A

ACVR2B
ACVRL1
ADCYAP1R1
ADIPOR1
ADIPOR2
ADRB2
AGTR1
AGTR2
AMHR2
ANGPT1
ANGPT4
ANGPTL1
ANGPTL3
ANGPTL4
ANGPTL6
APLNR
AR
AVPR1A
AVPR1B
AVPR2
BMPR1A
BMPR1B
BMPR2
BRD8
CALCR
CNTFR
CRHR1
CRHR2
CRIM1
CRLF1
CRLF2
CRLF3
CSF1R
CSF2RB
CSF3R
ENG
EPOR
ESR1
ESR2
ESRRA
ESRRB
ESRRG
FGFR1
FGFR2
FGFR3
FGFR4

FGFRL1
FLT1
FLT3
FLT4
FSHR
GALR2
GALR3
GCGR
GHR
GHRHR
GHSR
GIPR
GLP1R
GLP2R
GNRHR
GPER1
HNF4A
HTR3A
HTR3B
HTR3C
HTR3D
HTR3E
IFNGR2
IGF1R
IGF2R
IL10RB
IL12RB1
IL13RA1
IL13RA2
IL15RA
IL2RB
IL17RA
IL17RB
IL17RC
IL17RD
IL17RE
IL18R1
IL18RAP
IL1R1
IL1R2
IL1RAP
IL1RL1
IL1RL2
IL20RA
IL20RB
IL21R

IL22RA1
IL22RA2
IL23R
IL27RA
IL2RA
IL2RG
IL31RA
IL3RA
IL4R
IL5RA
IL6R
IL9R
INSR
KDR
LEPR
LGR4
LGR5
LGR6
LHCGR
LIFR
LTBR
MC1R
MC2R
MC3R
MC4R
MET
MLNR
MPL
MTNR1A
MTNR1B
NGFR
NMBR
NPR1
NPR3
NR0B1
NR0B2
NR1D1
NR1D2
NR1H2
NR1H3
NR1H4
NR1I2
NR1I3
NR2C1
NR2C2
NR2E1

NR2E3
NR2F1
NR2F2
NR2F6
NR3C1
NR3C2
NR4A1
NR4A2
NR4A3
NR5A1
NR5A2
NR6A1
NRP1
NRP2
OGFR
OPRD1
OPRK1
OPRL1
OPRM1
OSMR
OXTR
PGR
PGRMC2
PPARA
PPARD
PRLHR
PRLR
PTGER1
PTGER2
PTGER4
PTGFR
PTH1R
PTH2R
RARA
RARB
RARG
RORA
RORC
RXFP2
RXRA
RXRB
RXRG
S1PR1
S1PR2
SCTR
SDC1

SDC2
SDC3
SDC4
SORT1
SSTR2
SSTR5
ST2
TACR1
TEK
TGFBR1
TGFBR2
TGFBR3
THRA
TIE1
TNFRSF10C
TNFRSF10D
TNFRSF11A
TNFRSF12A
TNFRSF13B
TNFRSF13C
TNFRSF14
TNFRSF17
TNFRSF18
TNFRSF1A
TNFRSF25
TNFRSF4
TNFRSF6B
TNFRSF8
TNFRSF9
TRHR
TUBB3
VIPR2
PTPN11
ICAM2
ITGAL
ITGB2
NCR2
LCK
FCGR3A
FCGR3B
NCR1
NCR3
CD247
ZAP70
LCP2
LAT

PLCG1
SH3BP2
FYN
SHC2
SHC4
SHC3
SHC1
SOS1
SOS2
ARAF
BRAF
RAF1
HCST
CD48
CD244
PRKCA
SH2D1B
SH2D1A
FAS
GZMB
PRF1
CASP3
BID
CD3D
CD3E
CD3G
PTPRC
ITK
TEC
NCK1
NCK2
GRAP2
PAK2
PAK4
PAK5
RHOA
CDC42
CD28
ICOS
MAP3K8
MAP3K14
CTLA4
CBLC
CBL
PDK1
PRKCQ

TRAC
TRAJ1
TRAJ2
TRAJ3
TRAJ4
TRAJ5
TRAJ6
TRAJ7
TRAJ8
TRAJ9
TRAJ10
TRAJ11
TRAJ12
TRAJ13
TRAJ14
TRAJ15
TRAJ16
TRAJ17
TRAJ18
TRAJ19
TRAJ20
TRAJ21
TRAJ22
TRAJ23
TRAJ24
TRAJ25
TRAJ26
TRAJ27
TRAJ28
TRAJ29
TRAJ30
TRAJ31
TRAJ32
TRAJ33
TRAJ34
TRAJ35
TRAJ36
TRAJ37
TRAJ38
TRAJ39
TRAJ40
TRAJ41
TRAJ42
TRAJ43
TRAJ44
TRAJ45

TRAJ46
TRAJ47
TRAJ48
TRAJ49
TRAJ50
TRAJ52
TRAJ53
TRAJ54
TRAJ56
TRAJ57
TRAJ58
TRAJ59
TRAJ61
TRAV1-1
TRAV1-2
TRAV2
TRAV3
TRAV4
TRAV5
TRAV7
TRAV8-1
TRAV8-2
TRAV8-3
TRAV8-4
TRAV8-6
TRAV8-7
TRAV9-1
TRAV9-2
TRAV10
TRAV12-1
TRAV12-2
TRAV12-3
TRAV13-1
TRAV13-2
TRAV14DV4
TRAV16
TRAV17
TRAV18
TRAV19
TRAV20
TRAV21
TRAV22
TRAV23DV6
TRAV24
TRAV25
TRAV26-1

TRAV26-2
TRAV27
TRAV29DV5
TRAV30
TRAV34
TRAV35
TRAV36DV7
TRAV38-1
TRAV38-2DV8
TRAV39
TRAV40
TRAV41
TRBC1
TRBC2
TRBD1
TRBD2
TRBJ1-1
TRBJ1-2
TRBJ1-3
TRBJ1-4
TRBJ1-5
TRBJ1-6
TRBJ2-1
TRBJ2-2
TRBJ2-3
TRBJ2-4
TRBJ2-5
TRBJ2-6
TRBJ2-7
TRBV2
TRBV3-1
TRBV4-1
TRBV4-2
TRBV4-3
TRBV5-1
TRBV5-4
TRBV5-5
TRBV5-6
TRBV5-7
TRBV5-8
TRBV6-1
TRBV6-2
TRBV6-3
TRBV6-4
TRBV6-5
TRBV6-6

TRBV6-7
TRBV6-8
TRBV6-9
TRBV7-2
TRBV7-3
TRBV7-4
TRBV7-6
TRBV7-7
TRBV7-8
TRBV7-9
TRBV9
TRBV10-1
TRBV10-2
TRBV10-3
TRBV11-1
TRBV11-2
TRBV11-3
TRBV12-3
TRBV12-4
TRBV12-5
TRBV13
TRBV14
TRBV15
TRBV16
TRBV17
TRBV18
TRBV19
TRBV20-1
TRBV24-1
TRBV25-1
TRBV27
TRBV28
TRBV29-1
TRBV30
TRDC
TRDD1
TRDD2
TRDD3
TRDJ1
TRDJ2
TRDJ3
TRDJ4
TRDV1
TRDV2
TRDV3
TRGV9

TRGV8
TRGV5
TRGV4
TRGV3
TRGV2
TRGJP2
TRGJP1
TRGJP
TRGJ2
TRGJ1
TRGC2
TRGC1
TRAV6

-DEGs AND ImmPort
